# Health‐care professionals’ assessment of a person‐centred intervention to empower self‐management and health across chronic illness: Qualitative findings from a process evaluation study

**DOI:** 10.1111/hex.13271

**Published:** 2021-05-02

**Authors:** Kristin Heggdal, Joshua B. Mendelsohn, Natalie Stepanian, Bjørg Frøysland Oftedal, Marie Hamilton Larsen

**Affiliations:** ^1^ Lovisenberg Diaconal University College Oslo Norway; ^2^ College of Health Professions Pace University New York NY USA; ^3^ University of Stavanger Stavanger Norway

**Keywords:** chronic illness, empowerment, health intervention, patient education, person‐centred care, qualitative research, recovery, salutogenesis, self‐management

## Abstract

**Background:**

Person‐centred care (PCC) empowers patients to manage their chronic illness and promote their health in accordance with their own beliefs, values and preferences. Drawing on health‐care professional's (HCP’s) experiences implementing an empowerment‐focused, person‐centred intervention called the Bodyknowledging Program (BKP), we undertook a process evaluation that aimed to assess the impact on patient health and well‐being.

**Methods:**

We used individual in‐depth interviews and semi‐structured focus groups comprising n = 8 interprofessional HCP who facilitated intervention sessions with n = 58 patients situated in Norwegian specialist care sites. Content analysis was used to analyse the data and summarize major themes.

**Results:**

Health‐care professional interviews revealed four main ways in which the intervention operated in support of health‐related patient outcomes: (i) addressing the whole person; (ii) hope and affirmation; (iii) expanding recovery; and (iv) social support and revitalized relationships. The intervention provided new tools for patients to understand the social, emotional and physical impact of their illness. Health‐care professional reported new insights to facilitate patient engagement and to promote patients’ health.

**Conclusions:**

The Bodyknowledging Program facilitated patient engagement through the promotion of patient‐centred care while developing the patients’ ability to exploit their own resources for effectively managing their health within illness. The process evaluation supported the underlying theoretical basis of the intervention and was suggestive of its potential transferability elsewhere.

## 1 INTRODUCTION

The global ambition of ‘leaving no one behind’ has been adopted by all member states of the United Nations and is grounded in 17 sustainable development goals (SDGs), which together form an urgent call for all countries to take action in critical areas, including the global burden of disease. An important target is to reduce the number of persons who die prematurely of chronic illness.^1^ Beyond efficient treatment and care, health promotion interventions have been offered to prevent secondary conditions, increase opportunities to participate in activities of daily living and attain optimal health.^2^ Empowerment is a key dimension in health promotion and is defined as a process through which individuals gain broader control over the decisions and actions that affect their health. Empowerment is fostered by participation and requires the legitimization of lay knowledge and active patient participation in their health‐care encounter.^3^ Although empowerment could offer a path to enhance clinical practice in order to improve the health and well‐being of patients diagnosed with chronic illness, there is a lack of interventions that utilize patients’ lay knowledge of their health and a lack of interventions that may be applied across diagnoses.^4^, ^5^, ^6^ Moreover, the World Health Organization ^7^ emphasizes the importance of person‐centred care (PCC) to promote better health outcomes and improve well‐being. The purpose of PCC is to empower patients to improve and manage their own health in accordance with their beliefs, values and preferences.^8^ In this study, the aim was to explore health‐care professional's (HCP’s) assessment of patients’ experience of engaging in an empowerment‐based, person‐centred intervention for patients with a variety of chronic illness in order to improve the delivery of the intervention.

### 1.1 Theoretical background

The Bodyknowledging Program (BKP) is an example of PCC.^9^ BKP is grounded in Bodyknowledging theory,^10^, ^11^ which asserts that people living with chronic illness possess bodily knowledge regarding their limits of tolerance concerning the type and magnitude of activity and factors in their physical (ie food or air quality) and psychosocial environment (ie significant others) that constitute an important resource for coping, recovery and health.^11^, ^12^, ^13^ This theory draws on Merleau‐Ponty´s phenomenological theory^14^ of the body as a foundation for knowledge and existence and Antonovsky´s^15^ theory of health as a dynamic continuum. In keeping with Paulo Freire's^16^ ‘pedagogy of the oppressed’ in which dialogue serves as the main method for helping people to understand their situations and to act in new ways, the essence of the BKP is to present patient‐centred expertise to be interpreted and applied by new patients. The BKP intervention aims to support the person's understanding of how they can utilize their inherent resources to handle the consequences of chronic illness, prevent deterioration and facilitate recovery and health within their specific life situation. Two main conceptions of recovery can be distinguished in the literature: ‘medical’ or ‘clinical’ recovery, referring to cure of an illness; and ‘personal’ or ‘life’ recovery, referring to a process of personal growth and health‐related change. In the context of chronic illness, personal recovery is not the same as being cured and/or having no further symptoms. Instead, it includes a ‘return to a state of wellness’ (eg following a relapse).^17^, ^18^, ^19^ This aligns with an understanding of health as a dynamic continuum as described by Antonovsky.^15^ In his theory, health is constituted by the sense of coherence (SOC), consisting of the dimensions; understanding, handling and meaning.^15^ The three dimensions in SOC are attended to in the BKP intervention.

In prior work, the intervention was piloted in both specialist and community health‐care settings in the south‐eastern region of Norway. Studies found that patients with a variety of chronic illness diagnoses reported that the programme allowed them to work systematically on their health as a process and enabled them to renew their participation in different areas of life, that is family life, working life and social life.^20^ In a separate study of the feasibility and outcomes in community care, participants reported that their engagement in the intervention improved perceived control of illness‐related stress and circumstances when HCP challenged patients to get to know their bodies and utilize their knowledge of health and illness.^21^ Another study investigating the efficiency of BKP in community care reported significant changes in recovery.^22^ A comparative study of BKP participants in specialist and community care showed that SOC improved at programme completion in both groups.^23^ Overall, there is promising evidence related to patient experiences and outcomes; however, the views and experiences of the participating HCP require further attention to further improve the process of implementation.

## 2 METHODS

### 2.1 The intervention

The Bodyknowledging Program (BKP) is designed to be broadly applied across diagnosis, age, gender and clinical settings and is organized into seven sessions and held in individual or group formats.^9^ Individual sessions lasted 1.5 hours. Group sessions last for 3 hours with a 30‐minute break and include 8‐10 individuals per group managing various health conditions (Table 1). Nurses, occupational therapists and physical therapists who had completed 80 hours of training (Table 2) lead the intervention groups. A poster, a flip‐over chart and a booklet/diary serve as pedagogical tools. Studying materials and sharing reflections on their health‐related challenges facilitate exploration, by the patient, of their recovery strategies while engaging in a supportive group process. In this way, patients’ own life situations, coping strategies and health‐promoting abilities form the core of the programme's content. In addition, patients were asked to choose a routine physical activity to complete at home twice a week in order to strengthen their physical capacity and facilitate reflection on their range of tolerance. Details on the development of the intervention and each intervention component have been published elsewhere.^9^

**TABLE 1:** Structure, aims and tools of the Bodyknowledging Program—a person‐centred intervention for facilitating empowerment, self‐management and health in chronic illness

Structure	Intervention aims and tools
Session 1	Programme introduction Introduction to programme structure, content and pedagogical tools. Introduction to the Bodyknowledging model. Tool: Bodyknowledging model
Session 2	Uncertainty Development of personal themes concerning living with chronic illness, with a focus on uncertainty and escaping and denying the sick body. Tools: Introduction to physical exercises, intervention booklet and diary.
Session 3	Loss of life space I Introduction to the phase of losing life space – grieving and anger. Development of personal themes concerning challenges with a long‐term condition and their health promotion strategies. Tools: Physical exercises, booklet and diary between and within sessions.
Session 4	Loss of life space II (In‐depth work) In‐depth work on personal themes concerning challenges with living with chronic illness. Acknowledging the body as a source of knowledge about health and illness. Understanding one´s limits of tolerance for activity and factors of the physical and social environment. Development of personal themes and strategies concerning health promotion in chronic illness. Tools: Physical exercises, booklet and diary
Session 5	Listening and understanding the body's signs I Exploring the body's limits of tolerance concerning the type and magnitude of physical activity (ie work, exercise), factors within the physical environment impacting health and type/magnitude of social relations impacting health (eg social network, significant others and professional relationships). Communicating limits of tolerance to others. Exploring the possibilities for extending limits of tolerances to facilitate wellness. Development of personal themes and strategies for creating health within illness. Tools: Physical exercises, booklet and diary.
Session 6	Listening and understanding the body's signs II (In‐depth Work) Getting to know one's body's capacity. Introduction of the phase of integrating embodied knowledge and exploring new possibilities for health. Living with chronic illness and encountering significant others, professional relationships and society. Development of personal themes of health promotion in chronic illness. Tools: Physical exercises, booklet and diary.
Session 7	Integrating knowledge—New possibilities for well‐being and health Summary of the Bodyknowledging model and personal application. Patients encounter with significant others and the society while living with chronic illness. Development of actions to sustain and strengthen health after programme completion. Tools: Physical exercises.

**TABLE 2:** Training programme for health‐care professionals (HCPs) leading the Bodyknowledging Program (BKP)

	Content
Course 1. Health promotion processes	
Course 1 has two 3‐day meetings (1 in‐person and 1 online) Method of work: Resource lectures are arranged based on the central topics. Written report 1 for study group with oral presentation during videoconference. Written report 2, individually, with guidance from supervisor.	Fundamental perspectives in health promotion work The resource perspective in meeting with people who live with health problems or who are at risk of being afflicted. Salutogenic theory, theory of coping and the application of the theory in practice.
	Health promotion processes Recovery research on long‐term illness within somatics and psychiatry. The health promotion process: Bodyknowledging and patients/users experiences with health promotion processes.
	Empowerment and user participation. Patients as experts on their health and how to handle health problems. Tools and making accommodations for user participation in practice.
	The significance of social relations for coping and health. Social networks and social context; inclusion and social interaction that promotes healing.
	Health education Dialogue as a fundamental approach in health promotion work. Narrative method, storytelling and writing as a method in health education. Group methods and group processes.
Course 2: Bodyknowledging as a process‐oriented approach to coping and health	
Course 2 has three 3‐day gatherings (1 in‐person and 2 online) Method of work: Written report 3: plan for carrying out the BKP in practice. Practical training on implementation of the BKP. Written report 4: after meeting with patient/group. Individual process reports from practical training. Instructor evaluation of competencies.	Bodyknowledging as an educational health system and tool for health promotion Structure, contents, methods and tools of the Bodyknowledging Intervention Program. Resource use and requirements for organization and quality. Interdisciplinary collaboration. Documentation of change throughout the process. Reflection upon own practice. Bodyknowledging Program as an interactive tool and follow‐up programme in the specialist and municipal health services. Bodyknowledging Program organized as a coping course, tool for individual tailoring of care and prevention, rehabilitation measures and preventive measures. Bodyknowledging Program compared with other educational health systems.
	Health education Solution‐focused approach to health promotion work. The physiotherapeutic method of Basic Body Awareness. Dialogue, storytelling, writing, solution‐focused therapy and exercises inspired by the physiotherapeutic method of Basic Body Awareness in implementation of the Bodyknowledging Program.

The present analysis was part of a larger study in which the BKP was piloted in different clinical sites.^20^, ^23^ In line with the recommendations for the development of complex interventions,^24^ a qualitative process evaluation^25^ was undertaken to identify key intervention components and the connections between intervention activities and outcomes. The method also served to examine the applicability of the theory underpinning intervention design.^26^ Qualitative data were collected using focus‐group and individual in‐depth interviews with HCP.^25^, ^27^ The Ethics Committee of the South‐eastern Regional Health Authorities in Norway approved the study.

### 2.2 Recruitment

The participating HCP discovered the Bodyknowledging model when attending a health conference in Norway and requested further training. Nine group facilitators were purposively selected in order to ensure interdisciplinary participation from a variety of health professions. All the facilitators were invited to participate in evaluation interviews. One participant was unable to complete the training, and pre‐training data were excluded from the analysis. Patients were interviewed from the same groups run by the facilitators. Results from patient interviews have been published elsewhere.^20^, ^22^, ^23^

Health‐care professional facilitators included four nurses (RN), three occupational therapists (OT) and one physical therapist (PT) with a range of 5‐35 years of experience in health care and who represented three different clinical sites in specialist care: an inpatient rehabilitation unit (n = 5), an outpatient clinic (n = 1) and an outpatient centre for patient education (n = 3). Table 3 summarizes characteristics of HCPs. Two HCPs, representing different health disciplines, led the intervention in pairs for 8‐11 patients diagnosed with a variety of chronic illnesses within each group (Table 4). The individual format was led by a nurse and was reserved for patients who had moderate cognitive disabilities, were depressed or did not want to participate in a group format. All HCP facilitators (n = 8) who completed the training and led the intervention at their clinical sites consented to participate in evaluation interviews as did patients involved in the study.

**TABLE 3:** Health‐care professionals by gender, age and clinical site

Participants	All HCP (N = 9)	Nurses (N = 5)	Occupational therapists (N = 3)	Physical therapist (N = 1)
Men	0	0	0	0
Women	9	5	3	1
Age range	36‐60	36‐59	44‐47	60
Years of experience	5‐35	10‐30	5‐20	35
Rehabilitation unit, Hospital 1 (inpatient)	6	3^a^	2	1
Outpatient clinic, Hospital 2	1	1	0	0
Learning and Mastery Center, Hospital 2 (outpatient)	2	1	1	0

^a^ One nurse did not complete the training due to sick leave.

**TABLE 4:** Patients by gender, age, diagnostic categories and site

N = 58	Group 1 LMC (N = 11)	Group 2 LMC (N = 9)	Group 1 Rehab (N = 8)	Group 2 Rehab (N = 10)	Individual format Rehab (N = 13)	Individual format outpatient Clinic (N = 7)
Men	6	3	8	6	9	2
Women	5	6	0	4	4	5
Age range	56‐68	29‐69	48‐89	46‐66	23‐81	31‐64
COPD (N = 4)	2	2				
IBD (N = 8)		1				7
Stroke (N = 18)			6	3	9	
Multiple sclerosis (N = 2)				2		
Neurological disease (N = 1)				1		
Head injury (N = 1)			1			
Brain tumour^a^ (N = 2)				1	1	
Cerebral atrophy (N = 1)					1	
Leg amputated (N = 3)			1	2		
Spina bifida (N = 2)				1	1	
Musculoskeletal pain (N = 5)	1	3			1	
Heart disease (N = 4)	4					
Diabetes (N = 3)	2	1				
Depression (N = 1)		1				
No diagnosis (N = 3)	2	1				

Note

Abbreviations: LMC, Learning and Mastery Center; COPD, Chronic Obstructive Pulmonary Disease; IBD: Inflammatory Bowel Disease.

^a^ Rehab after surgery.

### 2.3 Data collection

The first author conducted four focus groups and two individual interviews with the HCP lasting 60‐90 minutes in duration, 1‐2 weeks after the intervention was completed. Two focus‐group interviews (n = 8) were conducted with all participants from all three sites together. One focus group (n = 5) was conducted at the rehabilitation unit, and one was conducted at the centre for patient education (n = 2). Finally, two individual interviews were conducted with one nurse who worked individually with patients in the outpatient clinic. A semi‐structured interview guide was developed to guide the interviews and focus groups with questions such as: What are your thoughts on the usefulness of BKP to facilitate empowerment, self‐management and health in chronic illness? Did you observe any turning points or health‐related results for patients? If so, what do you think facilitated these?

### 2.4 Analysis

The interviews were audiotaped and transcribed verbatim. The first author and a research assistant analysed the data from each site independently and discussed the preliminary findings. The data were then analysed across sites. In order to ensure confirmability and dependability,^28^ findings were discussed in co‐operation with the whole research team. The evaluation focused on the HCP’s assessment of patient outcomes in relation to intervention activities. Patton`s^25^ description of ‘structure’, ‘process’ and ‘results’ served as the main headings for the analytic process. Each interview was read through several times to obtain a sense of the whole. Content analysis^29^, ^30^ was used for interpreting the data through a systematic process of coding and identification of themes. The parts of the text that described the HCP’s assessment of patient outcomes were extracted, and the text was divided into meaning units that described similar content. These were abstracted into themes and subthemes and labelled with a code. Themes, subthemes and codes were sorted, discussed and studied again in order to develop and report on general themes.

## 3 RESULTS

Health‐care professional assessments of BKP and patient‐reported outcomes were captured in four major themes: (i) addressing the whole person and individual needs; (ii) hope and affirmation; (iii) expanding recovery; and (iv) social support and revitalized relationships.

### 3.1 Addressing the whole person and individual needs

Health‐care professional reported that the new BKP approach brought the patients beyond the specific problems they experienced in the moment towards a focus on their life situation as a whole and their future life's unfolding. They elaborated that patients had found a space within the intervention to tell their life history and find new meaning in life post‐diagnosis. They felt that it was a great advantage to be trained to encourage patients to convey exactly how they were and ensure the importance of the patients’ experience. HCP reported that the intervention helped begin a process of repairing the disruption in their biography associated with the onset and progression of their illness.

…we get the possibility to focus on the whole person. In the start, patients are focused on the arm that is not functioning or their capacity for ADL, and they are focused on physical exercise. In BKP, we motivate them to go out and experience their daily life, to focus on the unfolding of their life, to get back to a life where they had lots of good experiences, and then, indirectly, this has an impact of the whole body, their coping and their capacity for endurance (PT).

Health‐care professional emphasized that the intervention and tools facilitated their work with patients over time with the aim of strengthening their capabilities for health. Helping patients to understand their health as a process and the possibilities of using their bodily knowledge consciously was integrated within this realization. However, HCPs emphasized that the intervention functioned dynamically insofar as ‘Bodyknowledging is the patients’ process’ and that the programme worked differently in relation to individual patients:

All the questions are not relevant for all patients at all times. It depends on where they are at the moment, what they are ready to attend to. I think that people are touched if it turns their attention to something that means something to them. They just pass the other questions that does not fit to their individual situation (OT).

Health‐care professional conveyed that the BKP appeared to be especially relevant to patients who can feel in their body that they have symptoms and the limitations of chronic illness on their life's unfolding. This includes patients who experienced symptoms and limitations but who did not yet have any diagnosis. They told about a man who did not dare to go out because he was too uncertain and no one could explain his condition.

There was a man in the group who had a lot of symptoms but no diagnosis. He recognized his own experience when he read the booklet with the examples from former patients in BKP. After some sessions, he argued that it was not so important for him to be diagnosed after all because now, he had got help and was able to live with the symptoms. (OT).

Health‐care professional argued that the intervention was especially useful in the transition period from hospital to home and as a community‐based health intervention.

Health‐care professional learned to identify programme boundaries. HCP underscored that the BKP may not fit patients who have a chronic illness without symptoms or who do not recognize their symptoms, that is a well‐regulated diabetes or patients with early stage of anorexia nervosa. Likewise, patients who were depressed and needed additional support may not benefit. Finally, HCP emphasized that patients in the acute phase of illness may not benefit as much as those who have some experience living with chronic illness.

### 3.2 Hope and affirmation

Health‐care professional reported that the phased approach to the intervention, reflecting a progression in the patients’ understanding of their illness, created hope and empowered patients to be taken seriously as the expert on their own illness experience.

The program allowed for a focus on coping which is founded on knowledge of different phases one naturally has to go through and the fact that it is possible to live with the condition, that there is hope. That it is possible to gain power and resources from within oneself and in the social network (RN).

Patients’ engagement facilitated improved communication regarding the application of their own language and knowledge to their illness experience. Health‐care professional observed that patients used the intervention model as a point of reference to compare their own experiences, and to assess where they were at the moment, discover something new about themselves and reflect on how they could move forward in their health‐promoting process. When asked to describe the outcome for patients, a nurse HCP referenced a male patient from the rehabilitation unit:

Before, it was a feeling I had. In this group, I have been able to find the words for describing it. Now, it has become knowledge, *my* knowledge. It has become clearer to me what I can and cannot do and what I want to and do not want to do. I can be much clearer in telling others too. Now, I know what is smart for me, and I can express this in a much clearer way to people around me (RN).

The nurse argued that the patient had received affirmation that his thoughts and feelings were valued inputs to his own recovery and this had made him less angry and more co‐operative with his family. She added that this clarified her practice of the intervention as a novel treatment in her encounters with patients diagnosed with chronic illness.

### 3.3 Expanding recovery

In BKP, former patient's experience of recovery and health within illness is described in the pedagogical tools. Therefore, patients were invited to review content in between sessions and were encouraged to engage in ‘recovery work’, including reading training materials and doing physical activity twice per week. Health‐care professional reported that patients’ use of the intervention booklet and diary increased as the sessions progressed. However, it was difficult to know whether the physical exercises they completed at home were conducted as planned. The HCP reported some activity results such as patients who had started to do occasional workouts. For example, a female participant started a swimming course, while others reported that they were ‘participating more in life again.’

Health‐care professional reported that a male participant who had a bad shoulder for 10 years realized that he had not considered that he was holding tension in his body. He reported that he was better able to relax post‐intervention. Health‐care professional relayed that a female participant reflected after the 2nd session: ‘Now, I understand that I have not been accepting the illness’. Health‐care professional emphasized how these patient reports facilitated the experience of progressing to normality.

Health‐care professional also observed that some patients were able to ascertain expanded comfort zones for their bodies, beyond their initial (pre‐intervention) understanding of their own physical limits. They (HCP) reported that one patient who experienced symptoms but had no diagnosis recognized himself within examples of other patient experiences described in the intervention booklet. After programme completion, he had realized that he could live with the condition, and he started to go to the grocery store, something he had been unwilling to do given his uncertainty about his body's limit of tolerance. Health‐care professional expressed that the intervention was useful even for patients who had lived with the illness for a long time without expanded physical capability because they were able to achieve some empathy for themselves, and to realize that they were allowed to be sad and that there was a grieving phase that may be complex and on‐going. In addition, Health‐care professional described how the intervention facilitated peer support; that is, when someone was describing their grief, others reacted with empathy.

We saw the effect of having people who described themselves as being healthy in spite of their illness, how they were able to support others who were dominated by grieving or anger due to their losses…After some sessions, we experienced that patients started to challenge each other in the group, to ask each other questions and ‘push’ each other a bit…and when one of them starts to open up, others also feel that it is alright to share their story (OT).

Health‐care professional described how the group process facilitated recovery and how the pedagogical tools served the assessment and identification of individual needs for follow‐up. They argued that even if some patients had difficulties expressing themselves, ie due to moderate cognitive deficits, they benefitted from listening to the others who shared their story and by participating in a safe group process according to their level of functioning.

Participants manage to place themselves in a bigger frame, to move the focus from one’s individual perspective to the people around them in the group, and not only focus on themselves, and to get feedback. I think this makes a big impression (RN).

However, HCP reported that some participants who had been socially isolated for a long time due to their illness attained a better outcome when engaging in individual sessions as this allowed for more openness to specific challenges and in‐depth work on their health. Health‐care professional emphasized the importance of holding on to the recovery perspective in patient encounters:

I think that it is very important that HCP do not put restrictions on the patient’s recovery, because we hear about this on and on again, that patients have got the message that they will not recover and that this (the illness and the consequences) is something they have to accept (RN).

### 3.4 Social support and revitalized relationships

One of the aims of BKP is to prepare people living with illness to re‐encounter social relations and to re‐engage in the social world. The mixed‐gender group was emphasized by HCP as a strength in this regard. They observed that patients began to challenge each other as the intervention progressed and to ask questions of each other. This helped patients focus on expressing themselves in public. Health‐care professional expressed the hope that patients’ experiences in the group could ameliorate their relationship quality and broader social interactions because important themes for the promotion of health and quality of life emerged within intervention groups that could be communicated onwards to key sources of social support, that is family and friends. Health‐care professional observed that participants ability to communicate had been strengthened, especially when it came to complicated feelings of grief and anger connected to their reduced capabilities relative to the period prior to their illness. This example was given by a nurse regarding a male participant who self‐described as modest:

… he dared to tell how he was and to talk with the other group members. And then, he told that he was struggling with the communication with his wife because he was afraid of getting negative feedback. I challenged him to talk to his wife because the feeling he had inside did not contribute to his recovery, and then he talked to his wife and he did not get the negative answer that he was prepared for…He got the affirmation that he was able to express his thoughts and feelings (RN).

Another HCP reported that a male participant who engaged in individual sessions expressed that the experience was clarifying for him in relation to the amount of work he could do while remaining well. Health‐care professional also reported that some patients revealed that their partner or daughter had read the intervention booklet, which resulted in mutual reflection on key questions related to recovery, and that this had a positive impact on their relationship. These findings suggested that the intervention assisted with strengthening the social support network by developing new insights that were communicated effectively within the group and beyond.

## 4 DISCUSSION AND CONCLUSION

We have presented an evaluation of the process of implementing an empowerment‐focused, person‐centred intervention called the Bodyknowledging Program (BKP) by assessing the impact on patients according to qualitative observations by HCP drawn from nursing, physical therapy and occupational therapy professionals. We found that HCP assessed that this intervention approach empowered patients to focus on their current life situation as a whole and their future life's unfolding. This was attained through promoting an understanding of the value of the patients’ own illness experience, the acceptance or expansion of the patients’ own physical capabilities, and the enhancement of positive social interactions. According to HCP in our study, the intervention served to reorganize ‘the disruptive experiences of chronic illness, in re‐ordering its arbitrary and threatening characteristics’^31^ (p. 179) while helping to initiate a new biographical chapter within a set of life opportunities differently understood. Person‐centred, empowerment‐focused interventions attempt to place this reordering in the hands of patients themselves. However, patients may be overwhelmed with the stress and consequences of illness and may need support from HCP in this regard. Our findings suggest that the intervention model itself may have created a focal point for expressions of support within the BKP groups and that important health transition processes were facilitated as the participants gained access to support from peers and HCP within the intervention's structure and content.^32^, ^33^ These findings also demonstrate how the theoretical framework^9^, ^11^, ^34^ and programme function to establish a new possibility for dialogue in health care between patients and HCP that emphasizes patient participation in their own treatment, rehabilitation and health promotion.

Health‐care professional in our study clearly observed the potential for patients to positively engage with the group and individual sessions towards improving key markers of good health. Health‐care professional described that the individual format allowed for in‐depth work on difficult stages in each person's process in order to help the person to move forward in their effort to manage the illness. However, HCP emphasized the great value of the group format in order for the patients to hear and recognize themselves in peers and to gain access to role models and ideas on strategies for recovery.

Health‐care professional’s assessment of patients’ experience, including hope, affirmation, extended recovery, social support and revitalized relationships, aligns with previously reported patient outcomes.^20^, ^22^ Prior findings in Norwegian rehabilitation and outpatient clinical settings found significant improvements in patients’ ability to manage their chronic illness.^20^, ^21^, ^23^ The patient‐reported outcomes were supported in this study by means of data on HCP’s assessment of patient experience following implementation of BKP. According to HCP, the intervention offered a space for patients to discover and take advantage of their inherent and often under‐utilized health resources such as their bodily knowledge on what makes their illness better or worse.^11^, ^12^, ^13^, ^35^ Individuals reflected and explored their individual strengths, resources and knowledge of their disease, which are necessary skills for self‐care management.^21^, ^23^ In addition, the BKP represented a possibility to see oneself from the point of view of others, and to gain access to other participants’ experience‐based knowledge of health. The benefit of peer support, sharing understanding and mutual learning and increase in hopefulness was reported as an important outcome in a scoping review of patient education programmes aimed at promoting self‐management for people with chronic illness.^5^

A lack of trust in patients’ experience‐based knowledge in chronic illness was suggested by Paterson^4^ as a challenge to patient empowerment in chronic illness. In the present study, HCPs were trained to affirm patients’ expertise and to challenge them to utilize their bodily knowledge of health^21^, ^23^ and observed that this approach facilitated patient engagement in knowledge about themselves and their health, and their ability to articulate their thoughts and feelings regarding their illness and recovery. Our findings are in accordance with a systematic review^36^ that found that a personalized care approach, which focuses on self‐management and the patient as a whole person, was associated with improvements in psychological health status and people's ability to self‐manage their condition. Self‐management involves handling emotional tasks, which require the capacity to deal with psychological responses. Parke^37^ suggested that current therapeutic interventions do not provide adequate support for individuals to self‐manage the emotional tasks and the psychosocial prerequisites for handling the consequences of chronic illness. Health‐care professional’s assessment of patient experiences in this study and patient‐reported outcomes in prior studies suggest that interventions that attend to this missing component may increase chances of success. One explanation for these observations is the progressive time frame of the intervention as patients gained access to contact with the same HCP over a longer period of time who were working along the lines of a strength‐based^38^ wellness and recovery perspective on chronic illness care.^39^

Health‐care professional observed patients’ personal growth and health‐related change process while engaging in BKP including a ‘return to a state of wellness’.^17^, ^18^ These observations align with the concept of ‘personal’ or ‘life’ recovery described in other studies.^39^, ^40^ To strengthen recovery, HCP emphasized the importance of acquiring tools for addressing the individual needs of the whole person in order to facilitate recovery. Health‐care professional reported that the intervention facilitated patient engagement in knowledge about themselves and their health and, importantly, by avoiding ‘demands’ for compliance. This may be contrasted with traditional approaches to patient education and self‐management,^41^ which focus more on ‘expert disease management’. By contrast, HCP reported that empowerment and individual health processes were kept at the forefront, pushing the illness to the background in order to open up a space for patients to take new steps towards recovery.^42^ Health‐care professional argued that the training was fundamental in order to focus on recovery and health instead of merely on disease and to relinquish the power embedded in their professional expertise that is at odds with the patient‐centred approach.^43^, ^44^, ^45^

Overall, these findings suggest that the BKP may be a broadly applicable tool for HCP to incorporate PCC and an empowerment‐based approach to health promotion in chronic illness.^34^

### 4.1 The study's strengths and limitations

Key strengths of this study include the focused aim, the use of theory to inform the intervention and the rich data set collected from key informants that were analysed across formats and clinical sites.^46^ Health‐care professional participating in this study took the initiative to develop the intervention, participated in the formative research and engaged in evaluation interviews. This may be regarded as both a strength and as a limitation. While a high level of clinical relevance was apparent, HCP participated in the evaluation of their own work with patients. However, other studies of the efficacy of the BKP with patients unaware of the background development have established the relevance (and scalability) of the BKP.^21^, ^22^ Given that health‐care professionals were trained on the underlying theory of the intervention, it was not clear to what extent their observations on patient experiences were reflecting underlying arguments and conceptualizations from the theoretical framework delivered during the training they attended. However, the training was identified as an important prerequisite for this kind of empowerment work. Four focus‐group interviews and two individual interviews were necessary to establish a rich data set on the HCP’s experiences of implementation of BKP in each individual site. Focus groups may have some disadvantages in the sense that the data collected are based on the social interaction and ‘knowledge construction process’ in the group and, hence, may not represent the individual views of participants.^27^ This limitation was mitigated by means of validation in individual interviews and by conducting interviews across sites. An additional strength of this work was the strong theoretical underpinnings of the intervention, which is suggestive of transferability to other contexts and settings.^26^, ^47^, ^48^ Future work should build on these findings and use the population, the intervention and the environment in the primary context^48^ as a backdrop while adapting and testing the intervention in other contexts.

## 5 CONCLUSION

The study assessed HCP’s report of the patients’ experience after facilitation of the Bodyknowledging Program (BKP), a person‐centred, empowerment‐focused intervention that aimed to supporting individuals in managing their chronic illness towards advancing the promotion of health and well‐being. Analysis of interviews with HCP suggested that the programme facilitated patients’ ability to explore their internal and external resources for health that are critical in their ability to manage their own care. Health‐care professional reflections in this work demonstrate their use, in practice, of a de‐medicalized framework that enabled their focus to land on the whole patient, their values and context. These findings suggest that evaluating the intervention elsewhere using robust study design and with careful attention to local settings and contexts is feasible and will provide benefits for the clinical practice of HCPs.

## 6 PATIENT OR PUBLIC CONTRIBUTION

Health‐care professionals (HCPs) were involved in the development, implementation and evaluation of the intervention, in discussions and in dissemination of the findings. Patients were involved in evaluation of the intervention.

## CONFLICT OF INTEREST

No conflict of interest.

## AUTHOR CONTRIBUTION

KH originally conceived the study, collected and analysed the data and prepared the first draft of the manuscript. JBM, NS, BFO and MHL contributed to revising it critically for important intellectual content.

## Data Availability

The data that support the findings of this study are available from the corresponding author upon reasonable request.
